# Leveraging Prior Information to Detect Causal Variants via Multi-Variant Regression

**DOI:** 10.1371/journal.pcbi.1003093

**Published:** 2013-06-06

**Authors:** Nanye Long, Samuel P. Dickson, Jessica M. Maia, Hee Shin Kim, Qianqian Zhu, Andrew S. Allen

**Affiliations:** 1 Center for Human Genome Variation, Duke University School of Medicine, Durham, North Carolina, United States of America; 2 Department of Biostatistics, Roswell Park Cancer Institute, Buffalo, New York, United States of America; 3 Department of Biostatistics and Bioinformatics, Duke University School of Medicine, Durham, North Carolina, United States of America; University of British Columbia, Canada

## Abstract

Although many methods are available to test sequence variants for association with complex diseases and traits, methods that specifically seek to identify causal variants are less developed. Here we develop and evaluate a Bayesian hierarchical regression method that incorporates prior information on the likelihood of variant causality through weighting of variant effects. By simulation studies using both simulated and real sequence variants, we compared a standard single variant test for analyzing variant-disease association with the proposed method using different weighting schemes. We found that by leveraging linkage disequilibrium of variants with known GWAS signals and sequence conservation (phastCons), the proposed method provides a powerful approach for detecting causal variants while controlling false positives.

## Author Summary

The decline in DNA sequencing cost permits the interrogation of potentially all variants across the entire allele frequency spectrum for their associations with complex human diseases and traits. However, the identification of causal variants remains challenging. Existing single variant tests do not distinguish between causal association and association induced by linkage disequilibrium and tend to be underpowered for rare or low-frequency variants, whereas variant grouping methods do not identify individual causal variants. We propose a novel Bayesian hierarchical regression approach that estimates effects of multiple variants on a disease trait simultaneously and incorporates prior information on the likelihood of causality. By simulation, we show that by combining linkage disequilibrium with known genome wide association signals and functional conservation, the proposed method, the first of its kind, is powerful to correctly detect causal variants.

## Introduction

Next-generation DNA sequencing technologies allow discovery of genetic variants across the full spectrum of allele frequencies, thereby enabling exhaustive screens for association between diseases and variants. Numerous statistical methods [1] have been developed for analyzing sequence variants. Recently, these methods have increasingly focused on rare variants because of their functional implications [2], [3], documented roles in disease etiology [4]–[7], potential contributions to missing heritability [8], and to the associations reported between common diseases and common variants [9]. Testing rare variants one at a time tends to have low power with a realistic sample size, especially in the presence of low penetrance and allelic heterogeneity (multiple variants at the same locus conferring risk). In an attempt to overcome this problem, grouping-based strategies have been proposed. These approaches typically involve grouping qualifying variants based on their location within a gene or pathway and testing the aggregated effect of the resulting set of variants. Several strategies for aggregating variant effects have been proposed: from simply collapsing rare variants [10] to summing weighted counts of minor alleles [11]–[13], and to more sophisticated approaches that attempt to incorporate the effect of both protective and neutral alleles [14]–[18]. Grouping-based testing remains an active area of methodological research and new methods continue to be developed. However, there are two significant limitations of any grouping-based test. First, the performance is critically dependent on the extent to which the grouping strategy reflects the genetic architecture of the disease being investigated. In order for grouping to be an effective strategy one must put ‘weight’ on those variants that are truly important. Putting ‘weight’ on unimportant or null variants will add noise to the statistic resulting in a loss of power. Since the genetic architecture of any disease is unknown, it is not always obvious which grouping strategy to prefer. Second, a significant grouping-based test implicates a genomic region or pathway and not specific genetic variants. Thus, the ultimate goal of identifying individual causative mutations remains elusive. For these reasons, some are reconsidering association testing of rare variation via grouping-based tests [19].

Here we introduce a Bayesian multi-variant liability regression model that does not involve grouping and tracks directly to individual variants. More generally, this model falls into a broader framework of Bayesian hierarchical modeling, which attempts to estimate all regression coefficients (variant effects) simultaneously by imposing variant-specific shrinkage on the estimated effects. This class of approaches has seen wide and successful applications in predicting quantitative traits, particularly in agricultural species [20]–[22]. However, its effectiveness in the analysis of sequence data in human disease studies is yet to be investigated.

There are two attractive features of the proposed approach. The first is that all variants are jointly analyzed so as to reduce bias of estimated effects by borrowing information from and/or accounting for other variants. The second feature is its ability to quantitatively incorporate prior information to weight individual variants based on their prior likelihoods of causality. Both features are expected to enhance power and reduce false discoveries. The weighting scheme is entirely customizable and can represent multiple sources of information. Here we have explored weighting schemes that incorporate two sources of information: sequence conservation (a natural measure of functionality of mutations [23], [24]), and linkage disequilibrium (LD) with significant SNPs detected by a genome wide association study (GWAS). The motivation to utilize LD between candidate variants and prior GWAS results is an attempt to mimic the real situation for most common diseases where large scale GWAS have been performed before any sequence data are analyzed and to effectively leverage that prior information. GWAS signals can guide the search for causal variants from sequence data both by defining candidate regions and by ‘tagging’ causal variants. The latter is the very assumption behind GWAS and has been recently shown to happen relatively often, even between rare causal variants and common tags [9].

We first tested the proposed Bayesian approach in fully simulated scenarios. We focused on causal variants at low and intermediate frequency (five MAF intervals between 0.2%–5%) and an intermediate genotypic relative risk (GRR) of 3 under a dominant model, which is when single variant tests would likely underperform given a realistic sequencing sample size. We evaluated performance by the power of the methods to correctly identify the precise causal variants (as opposed to association signals) and their false positive rates.

We then performed the Bayesian analysis on a set of exome sequencing samples, for which we assigned case/control statuses according to well-documented disease causing variants. We considered Crohn's disease and hepatitis C virus (HCV) treatment induced anemia. For Crohn's disease, there are two SNPs (rs2066844, MAF = 5.29%; rs2066845, MAF = 1.10%) in the *NOD2* gene that are known to be causal [25], [26]. Furthermore, GWAS run by the Wellcome Trust Case Control Consortium [27] found a signal (rs17221417, MAF = 25.41%) in the *NOD2* locus that is significantly (*p* = 9.4×10^−12^) associated with Crohn's disease. For HCV treatment induced anemia, rs6051702 was identified as the most significant signal (MAF = 17.81%, *p* = 1.1×10^−45^) in a GWAS scan [28], and in the same study, two functional variants (rs1127354, MAF = 7.55%; rs7270101, MAF = 13.85%) in the *ITPA* gene were also identified as causal. A subsequent biochemical study confirmed that these variants are indeed causal and described biochemically how they protect against treatment induced anemia [29]. Using these confirmed causal variants and GWAS signal for a given trait, we simulated case/control data from the available exomes, assuming heritability (on liability scale) = 10%, and assessed performance of the Bayesian approach.

## Methods

### Simulation study

#### Simulation of genotypes

The coalescent-based software GENOME [30] was used to generate a population of 15,000 haploid ‘genomes’ (effective population size ×2), each consisting of two 500 Kb-long independently assorting ‘chromosomes’. Mutation rate and recombination rate per base were set to 10^−8^ and 10^−8^ respectively. Causal variants were chosen within the central 100 Kb region of the first chromosome. To distinguish between the two chromosomes, the first was termed ‘causal chromosome’ and the second was termed ‘null chromosome’. Under this design, among variants being interrogated approximately a half of them were linked to causal variants to varying degrees and the other half were unlinked to any causal variant.

We simulated a three-locus dominant genetic model and a baseline disease risk of 1%. The presence of one or more causal (always minor) alleles increased the risk by a factor of GRR = 3. We were interested in the performance of the methods among different scenarios of causal variant MAF. We considered five MAF intervals: 0.2%–1%, 1%–2%, 2%–3%, 3%–4%, and 4%–5%.

#### Overview of the simulation study

**Figure 1:**
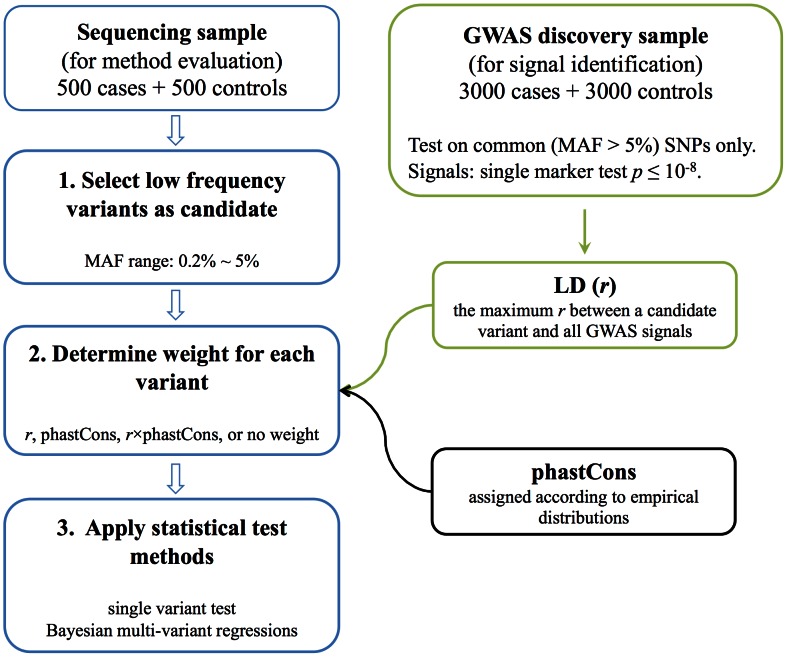
Workflow of the simulation study. Before carrying out these steps, a large pool of haplotypes (*n* = 15,000) was simulated. Given GRR and MAF of causal variants, cases and controls were simulated by randomly choosing pairs of haplotypes and calculating the risk of each individual to probabilistically assign phenotype.

A diagram of the procedure of analysis is shown in Figure 1. Briefly, a large GWAS cohort (3000 cases and 3000 controls) was simulated to identify significant association signals (*p*<10^−8^) and a smaller sequencing sample (500 cases and 500 controls) was used to detect causal variants among candidate variants.

We used a two-stage approach. In the GWAS stage, tests were limited to common SNPs (MAF>5%), since rare variants are typically underrepresented on GWAS arrays. On the other hand, in the sequencing data analysis, we focused on variants of MAF lower than 5% (step 1 in Figure 1), including the causal variants.

We considered two types of statistical methods for identification of causal variants. A univariate logistic regression model (step 3 in Figure 1), which tests one variant at a time, was used as a benchmark. In particular we fitted a genotypic model that typically results in a χ^2^ test with two degrees of freedom (or only one when the variant is too rare for minor allele homozygotes to be observed). This type of test cannot distinguish between causal variants and variants that are in LD with causal variants.

The Bayesian regression approach we implemented incorporates information from GWAS signals and sequence conservation (step 2 in Figure 1). We incorporated this information quantitatively into the Bayesian multi-variant model by assigning a weight to each candidate variant. Three types of weights were considered. First, based on the assumption that GWAS signals are in LD with at least one causal variant, the degree of LD between GWAS signals and a candidate variant is likely correlated with the likelihood of the candidate variant being causal. Therefore, for a given variant, we defined the ‘***r*** **weight**’ as the maximum of *r* values (square root of the LD measure *r* ^2^) between the candidate variant and all GWAS signals. Here, *r* was calculated using the 3000 GWAS controls (because LD is usually based on large control samples and can be obtained from the HapMap data set, for example). To prevent the *r* weights that enter the model from getting too small, we set a minimum value at 0.01 so that all *r* values less than that were forced to be 0.01. Second, we defined a composite ‘**phastCons weight**’ as the weighted average of phastCons among different lineages (1/2*vertebrateCons+1/3*mammalCons+1/6*primateCons) based on the consideration that conservation among a broader lineage scope implies potentially stronger functionality. phastCons scores for causal variants were drawn from the empirical distribution of ‘pathogenic’ SNPs in the dbSNP database; and phastCons scores for non-causal variants were drawn from the empirical distribution of all variants on chromosome 20 in a whole-genome sequence data set (161 samples) in our laboratory. As with *r* weight, the minimum composite phastCons value was set to be 0.01. Third, besides ‘*r* weight’ and ‘phastCons weight’ individually, we also calculated the product of the two, ‘***r***×**phastCons weight**’. Finally, an uninformative weight (all variants receiving the same weight, one, or ‘**no weight**’ as we called it) was also considered for comparison. All weights were scaled to be between 0.01 and 1 by dividing by the maximum.

#### Bayesian multi-variant liability model

In a liability model, it is assumed that a continuous latent variable (i.e. liability) underlies case/control status, and the liability follows a Normal distribution. A case (control) corresponds to a situation when an individual's liability is larger (smaller) than a threshold. A formal description is provided below.

Let *d_i_* and *y_i_* denote the disease status and liability, respectively, of individual *i* (*i* = 1,…,*n*); there is a fixed threshold *t* such that *d_i_* = 1 if *y_i_*>*t* and *d_i_* = 0 otherwise. Moreover, the liability is related to a set of variants by the Normal regression model:
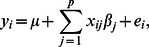
where *p* is the total number of variants; *μ* is the population mean; *x_ij_* is the genotype of variant *j* in individual *i*, coded as 0, 1 or 2 for aa, Aa or AA, where A is the minor allele of variant *j*. The genetic effect of variant *j* is given by *β_j_*, an unknown quantity which we wish to make inference about. Finally, *e_i_* is the random error with a Normal distribution 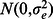. To ensure model identifiability, a standard choice is to set the threshold *t* = 0 and the error variance 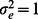.

In the above-mentioned liability model, the observed data consists of the binary response variables *d_i_* (*i* = 1,…,*n*) and genotypes *x_ij_* (*i* = 1,…,*n; j* = 1,…,*p*); the unknown parameters consist of *y_i_* (*i* = 1,…,*n*), *μ* and *β_j_* (*j* = 1,…,*p*). The central idea of a Bayesian analysis is to combine what is known about parameters before data are observed (represented by a prior probability distribution) with the information coming from the data (represented by a likelihood function) to arrive at a posterior probability distribution. Once the posterior distribution is known, one can make inference on parameters of interest (in our case, *β_j_*'s) using an appropriate Markov chain Monte Carlo sampling method.

Our belief of a variant being causal *a priori* enters the Bayesian modeling process through the specification of the prior distributions of *β_j_*'s. That is, we assume a Normal prior for *β_j_*: *N*(0, *w_j_σ* ^2^), where *w_j_* is the predefined weight of variant *j*, as noted earlier. According to this distribution, the larger the weight, the more likely that the variant has an effect. To complete the posterior distribution, we also need to write the likelihood function as well as prior distributions for *μ* and *σ* ^2^. Following common practice, we choose a flat prior for *μ* (*p*(*μ*)∝1), and a scaled inverted Chi-square distribution for *σ* ^2^, with both the degrees of freedom (*υ*) and scale (*S^2^*) being equal to 0.1. The final joint posterior distribution (up to proportionality) can then be written as
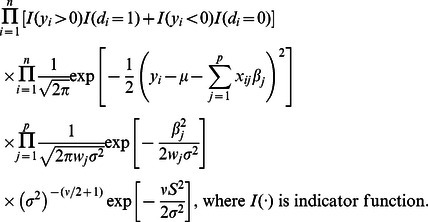
Using an iterative Gibbs sampler, one can draw values from each unknown parameter's conditional posterior distribution. Details regarding sampling procedure are outlined in Text S1. A single chain of Gibbs sampler was implemented, and the total number of iterations was 100,000 with the first 50,000 being discarded. After that, the chain was thinned by taking every 50^th^ value. Empirical evaluation of several random replicates of our simulated data showed that the posterior estimates of variant effects were robust to different initializations in the Gibbs sampling. For each variant effect, we recorded its posterior mean and standard deviation for significance test as described in the next section. A C++ program was coded for the Bayesian analysis. With a sample size of 1000 (500 cases and 500 controls) and a total number of 1000 variants, a complete run with 100,000 iterations took approximately 1 CPU hour on a 64-bit, 2.5 GHz Linux workstation.

#### Permutation-based significance test

Phenotype permutation was employed to determine an empirical significance threshold for statistically significant effects (causal). This method has been theoretically justified in controlling family-wise type I error [31], [32] and applied in multi-marker based QTL mapping [33]. The first step is to randomly shuffle case/control statuses across individuals while keeping their genotypes unchanged, in order to conserve LD structure. Suppose that the data are shuffled *B* times. The second step is to apply a statistical test on each of the *B* shuffled data sets and obtain a corresponding test statistic. For single variant logistic regression, we chose the minimum model *p*-value across all variants as the test statistic. For the Bayesian multi-variant regression, we chose the maximum absolute standardized effect size (the posterior mean of an effect divided by its posterior standard deviation) across all variants as the test statistic. The second step yields an empirical distribution of the test statistic under the null hypothesis of no variant-disease association. Finally, we compute a permutation *p*-value for each variant as (*B_0_*+1)/(*B*+1), where *B_0_* is the number of permutations that yield a test statistic at least as extreme as the one from unshuffled data. We used a significance threshold of *p* = 0.01 for all analyses throughout. Because of the computational burden with the Bayesian method and the need to replicate the study in different scenarios, we ran *B* = 500 permutations, which corresponded to a binomial standard error of *p* values of approximately 0.004 (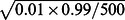).

#### Power and false positive rate

The entire procedure (data simulation and statistical analysis) was fully and independently repeated 200 times. Power of detecting a causal variant was the proportion of the 200 replicates in which it was declared to be significant. For non-causal variants, we calculated the proportion of them that were incorrectly identified as causal, i.e., the false positive rate (FPR). In each replicate, FPR was calculated separately for non-causal variants on the causal chromosome and those on the null chromosome. It was then averaged over the 200 replicates. Due to LD with causal variants, it may be inappropriate to call a non-causal variant on the causal chromosome a false positive if it was identified as causal. However, we loosely used the term FPR for convenience.

### Exome sequencing data analysis

#### Genotype data

753 individuals of European ancestry were whole-exome sequenced in our laboratory. A series of quality control (QC) filtering (including read depth, base calling quality, missing rate per SNP and per sample, genotype imputation accuracy, Hardy-Weinberg equilibrium test and MAF) were applied to remove SNPs and individuals. After QC, the *NOD2* data included 728 individuals and 100 candidate variants, and the *ITPA* data included 715 individuals and 338 candidate variants (Text S2). We next describe briefly the way we obtained these variants.

In the three well-known *NOD2* mutations on chromosome 16 for Crohn's disease, we focused on rs2066844 and rs2066845 only, because the third one (rs2066847) is an insertion variation and absent from our exome data. In addition, the GWAS signal in *NOD2*, rs17221417, was also unavailable in our data. However, we found another SNP, rs2066842, located 5 Kb downstream of rs17221417, is in complete LD with rs17221417 (both *r* ^2^ and *D*′ are equal to one as verified in HapMap data) and is present in the data, making it a perfect proxy for rs17221417. Attention was then restricted to 1 Mb region centered around the position of rs17221417. Within this region, variants with MAF≥0.2% were chosen as candidate (proxy SNP rs2066842 was excluded), resulting in a total of 100 variants. While the overall LD pattern showed small linkage disequilibrium among the variants, there were a handful of SNP pairs in high or even complete LD (1, upper panel). However, we did not observe SNPs in strong LD with the two causal variants in this region.

The two *ITPA* variants on chromosome 20 responsible for HCV treatment induced anemia, rs1127354 and rs7270101, as well as the associated GWAS signal (rs6051702) were dealt with in exactly the same way as the *NOD2* variants. A perfect proxy was used in lieu of the original GWAS signal, which was absent from our data. Within 1 Mb region centered around rs6051702, there were 338 variants with MAF≥0.2% and chosen for analysis. Inspection of LD plot (Figure S2, upper panel) revealed a few LD blocks among the 338 variants. In particular, there was one SNP in complete LD (*r* ^2^ = 1) with causal variant rs1127354 and two SNPs in almost complete LD (*r* ^2^ = 0.9) with causal variant rs7270101.

#### Phenotype simulation

To simulate case/control phenotypes, we started by generating a quantitative liability for each individual. Symbolically, *l* (liability) = *g* (genetic value)+*e* (error), as noted from our previous description of liability model. Assuming a dominance model as above, we set *g* = 1 when a risk allele (always minor) was seen at any of the two causal sites, and *g* = 0 otherwise. The error was drawn from a Normal distribution with mean zero and variance *V_e_*; value of *V_e_* was chosen such that heritability, *V_g_*/(*V_g_*+*V_e_*), was equal 10%, where *V_g_* was estimated by the empirical variance of *g*'s across samples. To achieve an equal number of cases and controls, we used the median of all samples' liability as a threshold so that a half of samples below the threshold were designated as controls and the other half as cases. Since only a small fraction of individuals carried risk alleles, the entire liability distribution was dominated by that of non-carriers. Also, as we assumed a low heritability (10%), the error variance was therefore large, leading to a wide overlap between liability distribution of carriers and of non-carriers (Figure S3). As a result, a small portion of carriers contributed to the control population whereas almost a half of non-carriers contributed to the case population.

#### Analysis

Since the genotypes were from real data and thus fixed, we were unable to assess statistical power and FPR systematically as in the full simulation. On the other hand, there will be uncertainty in the results from simulating only one set of phenotypes given the genotype data and applying the test method. To address this issue, we generated 100 sets of phenotypes using the same causal effects, giving rise to 100 replicated data sets, although each had the same genotype data. In each replicate, statistical analyses followed previous description for fully simulated data set, except that the number of permutations was increased to 1000. This way, ‘average’ performance of a method can be evaluated.

## Results

### Full simulation study

Across the 200 replicates, the number of candidate variants ranged from 862 to 1150, with a mean number 991. Each data replicate was analyzed by the single variant test (benchmark) and the Bayesian multi-variant liability regression model with ‘no weight’, ‘*r* weight’, ‘phastCons weight’, and ‘*r*×phastCons weight’.

As expected, increasing causal MAF led to an increase in the power of all methods (Table 1). Notably, even in the lowest MAF range (0.2–1%), the best method, Bayesian model with ‘*r*×phastCons’ weight, achieved an average (of the three causal variants) power of 0.34 and was able to detect at least one causal variant 67% of the time. When MAF was raised to 4–5%, the average power was 0.4 for single variant test, 0.7 for Bayesian models with *r* weight and no weight, and 0.9 for Bayesian models with phastCons and *r*×phastCons weight.

**Table 1:** Power of different methods in the simulation analysis.

Causal MAF	Number of causal variants detected	Single variant test	Bayesian w/o wt	Bayesian wt = *r*	Bayesian wt = phastCons	Bayesian wt = *r* × phastCons
0.2–1%	At least one^1^	0.120	0.010	0.085	0.220	0.670
	At least two^2^	0.005	0	0	0.070	0.310
	All three^3^	0	0	0	0.020	0.050
	Average^4^	0.042	0.003	0.028	0.103	0.343
1–2%	At least one	0.445	0.270	0.420	0.845	0.945
	At least two	0.085	0.020	0.075	0.590	0.685
	All three	0	0	0.015	0.260	0.285
	Average	0.177	0.097	0.170	0.565	0.638
2–3%	At least one	0.660	0.700	0.795	0.990	1
	At least two	0.180	0.220	0420	0.875	0.895
	All three	0.010	0.040	0.130	0.515	0.535
	Average	0.283	0.320	0.448	0.793	0.810
3–4%	At least one	0.795	0.885	0.935	1	1
	At least two	0.290	0.510	0.625	0.950	0.940
	All three	0.030	0.100	0.145	0.610	0.570
	Average	0.372	0.498	0.568	0.853	0.837
4–5%	At least one	0.835	0.980	0.990	0.995	0.995
	At least two	0.380	0.785	0.785	0.975	0.950
	All three	0.060	0.285	0.320	0.720	0.660
	Average	0.425	0.683	0.698	0.897	0.868

Results were based on 200 replicates. In each replicate, 500 cases and 500 controls were used to identify three causal variants from a total of ∼1000 variants, with each method being evaluated. We assumed causal variants have a constant GRR of 3 and render disease susceptibility under a dominant model.

1 The proportion of replicate simulations in which at least one causal variant was detected.

2 The proportion of replicate simulations in which at least two causal variants were detected.

3 The proportion of replicate simulations in which all three causal variants were detected.

4 The average power of three causal variants.

FPR was overall controlled at a very low level (Figure S4). Across all scenarios, the maximum averaged FPR was about 7 out of 1000, as produced by single variant test on non-causal variants on the causal chromosome. As expected, FPR on the causal chromosome was consistently higher than that on the null chromosome.

Next we describe in more detail results from the comparison among different methods.

**Figure 2:**
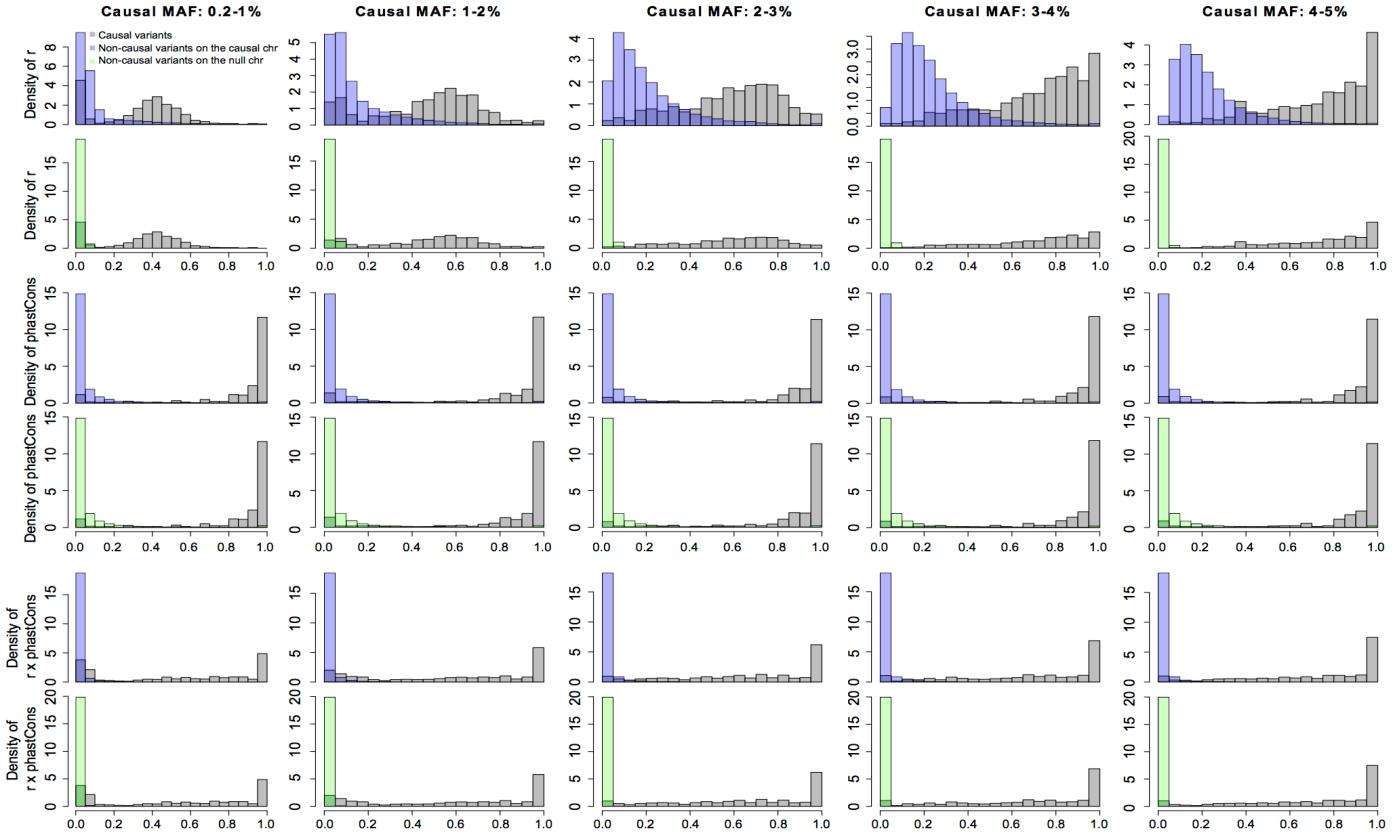
Distributions of three informative weights (*r*, phastCons and *r*×phastCons) for causal variants and non-causal variants on the causal and null chromosomes in the simulation study. In each MAF range, weights were collected from 200 replicates, and weights in each replicate were scaled by dividing each by the maximal value so as to bound final weight between 0 and 1.

1. **Bayesian multi-variant model with** ***r*** **weight consistently improved power over the one without weight.** The percentage of increase was especially appreciable in low MAF ranges. For example, the average power under MAF = 1–2% was 0.097 and 0.17 for no weight and *r* weight, respectively, corresponding to a 75% increase from no weight to *r* weight (Fisher's exact test *p* = 0.038). Likewise, under MAF = 2–3%, we found a 41% increase in power from no weight (0.32) to *r* weight (0.45) (Fisher's exact test *p* = 0.01). The advantage of using *r* weight was attributed to its discriminative ability between causal and non-casual variants (see the first two rows in Figure 2). Despite substantial overlap observed between *r* distributions for non-causal variants on the causal chromosome and for causal variants, their difference was rather distinct. Moreover, for non-causal variants residing on the null chromosome, the difference became much more profound, since *r* values of the vast majority of the non-causal variants with GWAS signals were close to zero. These indicate that causal variants were indeed preferentially in LD with GWAS signals [34] and, more importantly, such information is beneficial for detecting causal variants.
2. ***r*** **×phastCons weight greatly boosted power as compared with** ***r*** **weight alone.** As noted earlier, there was a substantial fraction of low *r* weight causal variants, especially at low causal MAF. This is because common GWAS signals cannot adequately tag a rare causal variant. Moreover, due to LD with causal variants, it is likely for some non-causal variants to receive *r* weights as high as those for causal variants, thereby further attenuating the contrast between causal and non-causal variants. Collectively, these results suggest that *r* alone is not informative enough and explain why it is underpowered at low causal MAF. On the other hand, phastCons appeared to distinguish causal variants from non-causal variants considerably better than *r* did and, by design, was independent of MAF. As a result, when using the product of *r* and phastCons as weight, the power was significantly boosted over *r* weight across all MAF ranges (Fisher's exact test *p* all smaller than 10^−5^).
3. ***r*** **×phastCons weight had higher power than phastCons alone when MAF<2% and similar power when MAF>2%.** Comparing phastCons against *r*×phastCons, it was found that both weights rendered the Bayesian model equivalent average power (about 0.8∼0.9) when causal MAF was greater than 2%. Below 2%, however, the advantage of *r*×phastCons was remarkable. In particular, when MAF was between 0.2% and 1%, Bayesian with the *r*×phastCons weight was markedly better powered than that with phastCons (0.34 *vs.* 0.10, Fisher's exact test *p* = 9.6×10^−9^). A closer examination of the distribution of effect sizes (first row of Figure S5) revealed that estimated effects of causal variants were generally larger with *r*×phastCons weight than with phastCons, especially for MAF between 0.2–1%. The increase of causal MAF resulted in increased effect sizes for both weights and, in the meantime, lessened their difference as well.
4. **Bayesian with** ***r*** **×phastCons weight outperformed other methods in controlling FPR**. As causal variants became more common, the degree of LD as measured by *r* between them and linked non-causal variants also increased, resulting in elevated FPR in general (first column of Figure S4). Single variant test could not control FPR well on the causal chromosome; many more false positives were discovered, particularly at higher causal MAF's. The same was found for Bayesian models with no weight and *r* weight. In contrast, Bayesian models with phastCons and *r*×phastCons weights were relatively more robust to causal MAF in maintaining FPR. This was most clear in the two highest MAF ranges (3–4% and 4–5%), where FPR by these two weights were substantially lower than that by other methods.
  We next examined FPR of non-causal variants on the null chromosome (second column of Figure S4). Not surprisingly, the average FPR of these null variants was close to zero by all methods. However, if we consider the proportion of the 200 replicates where false positives occurred, Bayesian model with phastCons weight appeared to produce the greatest FPR. We were particularly interested in the relative performance of *r* × phastCons *vs.* phastCons, and compared their effect estimates at these null variants (second row of Figure S5). While the estimated null effects were all concentrated around 0 distributionally, *r*×phastCons weight presented a much sharper peak around 0. There was a ‘heavier’ tail on nonzero values for estimated effects using the phastCons weight, making this method more prone to false discoveries. Such difference in the sizes of estimated effects between the two weights may be explained by the underlying distributions of the weights. Indeed, while the vast majority of phastCons (4^th^ row of Figure S6) of the null variants was concentrated on the smallest value (−2 on the log scale, or 0.01 on the original scale), there was a fair amount of probability mass within range −1 to 0 (0.1 to 1 on the original scale). In contrast, we found virtually zero mass for the *r*×phastCons weight (last row) beyond −1, and the entire distribution was shifted to the left as compared to phastCons weight. These results indicated that the incorporation of *r* into variant weighting suppressed false positives effectively, making *r*×phastCons weight the method of choice when considering both power and FPR.

In summary, by simulation we showed that Bayesian multi-variant liability regression model with informative weight assigned to variants substantially improved the power to detect causal variants, compared with single variant test and unweighted Bayesian model. In particular, we found that the product of *r* and phastCons constitutes a better weight than either alone in terms of power and FPR, especially at low causal MAF.

### Exome sequencing data analysis

We then applied our method to real exome sequence data with simulated phenotypes. Information about the two *NOD2* causal variants (rs2066844 and rs2066845) and the two *ITPA* variants (rs1127354 and rs7270101) is given in Table 2. In both data sets, while LD of causal variants with GWAS signal was higher than most of the non-causal variants, there existed non-causal variants with higher LD (Figure S1, S2, lower panel). Such occasional high LD with GWAS signal for non-causal variants was also observed in the previous simulated genomes data. Similarly, for some non-causal variants their phastCons scores were higher than those of causal variants. This is not surprising because variants not causal for one disease may be causal for other diseases. For example, in the *NOD2* data, 28% of non-causal variants had higher composite phastCons scores than the causal variant rs2066844 (0.32), and in the *ITPA* data 34% of non-causal variants had higher scores than the causal variant rs7270101 (0.17). However, it was rare for non-causal variants to have both high *r* and high phastCons. This made *r*×phastCons an attractive weighting scheme because it incorporates both measures to discriminate causal from non-causal variants.

**Table 2:** *NOD2* and *ITPA* causal variants in the exome sequencing data.

Gene	Causal variant	Chromosome	Build 37 position (bp)	MAF	LD (*r*) with GWAS signal	Composite phastCons score
*NOD2*	rs2066844	16	50745926	5.29%	0.39	0.32 (0.16, 0.24, 0.95)
	rs2066845	16	50756540	1.10%	0.16	0.99 (1, 1, 0.96)
*ITPA*	rs1127354	20	3193842	7.55%	0.34	0.99 (1, 1, 0.99)
	rs7270101	20	3193893	13.85%	0.58	0.17(0.016, 0, 0.95)

The composite phastCons is weighted sum of vertebrate cons, mammal cons and primate cons (shown in parenthesis), with weight 1/2, 1/3 and 1/6, respectively.

**Figure 3:**
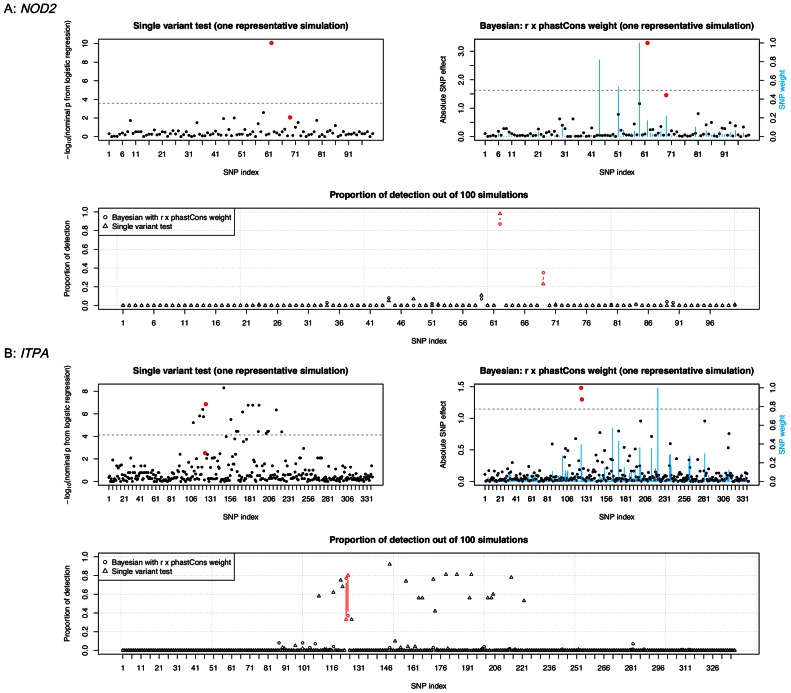
Causal variant detection in the exome sequencing data analysis. (A): *NOD2* data; (B): *ITPA* data. The two top panels are from one replicate of the simulation. For single variant test, SNP effect size was represented by −log10 of *p* value from logistic regression model; for Bayesian liability model, it was represented by the standardized effect estimated at each SNP. Red dots indicate two causal variants (see Table 1 for more information). Blue vertical bars show values of SNP weights (*r* × phastCons). The horizontal dashed line indicates effect size at the significance threshold (permutation *p* value = 0.01). The bottom panel shows proportion of simulations where a variant was detected (i.e., significant at permutation *p* = 0.01 level). Causal variants are marked in red color.

In Figure 3 (A) and (B), we first show a Manhattan plot from single variant test and one from Bayesian liability model with *r*×phastCons weight, based on one representative example out of the 100 simulated data sets. We then summarize results from both methods by displaying for each candidate variant the proportion of the 100 simulations where it was detected (i.e., being declared as significant).

As seen in Figure 3 (A), *r*×phastCons weights had a clear pattern in the *NOD2* sequence variants: the two causal variants ranked 4^th^ and 5^th^ among all 100 variants; with a few exceptions, almost all non-causal variants had very small weights. The last panel illustrates by replicating simulations how well each method identified causal *vs.* non-causal variants. For the relatively common causal variant rs2066844 (MAF = 5%), it was almost always identified by both methods across the 100 simulations. For the low-frequency causal variant rs2066845 (MAF = 1%), however, proportion of detection was about 40% and 20% by the Bayesian model and single variant test, respectively. Considering the low heritability (10%) and modest sample size (∼700), 40% is a substantial improvement over 20% for detecting causal variant at an allele frequency of 1%. In the meantime, false detection among non-causal variants produced by either method was negligible, likely a result of the absence of linkage disequilibrium in this region.

Compared with variants in the *NOD2* region, variants in the *ITPA* region had a less clear pattern in *r*×phastCons (Figure 3(B)): causal variant rs1127354 ranked at the 4^th^ whereas causal variant rs7270101 ranked at the 21^st^. The lower weight of rs7270101 had to do with the fact that it was an intronic SNP and was less conserved. While the Bayesian model outperformed single variant test for rs1127354 (MAF = 7.55%) by a large margin (80% *vs.* 30%), for rs7270101 (MAF = 13.85%) the pattern was opposite: the proportion of single variant far exceeded that of the Bayesian model (80% *vs.* 40%). However, the higher power of single variant test was accompanied by a large number of false positives, as opposed to virtually no false positives by the Bayesian model. This is in fact a strength of our Bayesian model in that it is able to select the correct causal variants out of many SNPs in LD. In addition, the two causal variants were always the top ranked variants in the Bayesian results while they were lower ranked in the single variant test. This makes prioritization much more accurate and efficient by the Bayesian model.

## Discussion

Here we developed and evaluated a Bayesian multi-variant regression approach for detecting causal variants in sequence data. We first tested its performance in simulated data using an intermediate risk (GRR = 3), a moderate and realistic sample size (500 cases and 500 controls), and a range of minor allele frequencies for the causal variants. Compared with the standard single variant test, our method, when coupled with informative prior weights, showed a clear advantage in statistical power. Application of the method to real exome sequence data in order to identify known causal variants implicated through GWAS showed similar results. In particular, while the high LD among variants in the *ITPA* data created great ambiguity in interpreting the results from single variant test, the Bayesian model using *r*×phastCons to weigh variants was able to effectively discriminate causal from non-causal variants in terms of their effect size estimates.

The Bayesian multi-variant regression model entails leveraging informative prior information on variants so as to better distinguish causal variants from non-causal variants. In this study, the uses of different kinds of weights give rise to different prior specifications (shrinkage) of variant effects, which in turn affect posterior estimation of variant effects and their statistical significance. As we have shown in the Results section, power and FPR of the Bayesian regression model were greatly dependent on the choice of prior weighting scheme. A good weighting scheme is data-dependent and often requires combining different pieces of information. In the full simulation study, which served as a proof of concept, we generated data assuming that variant causality is correlated with its conservation as encoded by phastCons. We found that the combination of significant associations from GWAS and variant phastCons rendered the Bayesian model a highly improved performance in power than one without weighting. However, while there is a tendency for causal variants to be evolutionarily more conserved for many diseases, this may not be always true, especially for late on-set diseases that are not subject to natural selection. In such scenarios, some form of prediction for variant effect, such as the PolyPhen scores [35] for coding variants would be a more appropriate functional measure.

Using prior biological knowledge in genetic association studies has increasingly been adopted recently. Several studies have proposed using variable significance threshold for variants. For example, the Prioritized Subset Analysis (PSA) [36] partitions variants to two subsets, a ‘prioritized set’ and a ‘complementary set’, where the prioritized set contains variants that are more likely to harbor causative loci. It then controls false discovery rate in each set separately, resulting in different thresholds on nominal p values for prioritized and complementary variants. The partitioning of variants into two different priority sets can also be iteratively optimized given the data [37]. Alternatively, a more sophisticated method is to have variant specific threshold or weight based on their functionality [38]. In addition, grouping-based association tests can also aggregate variants differentially according to some prior knowledge [12], [39]. One essential difference between our method and previous methods is that we fit all variants simultaneously while the variable threshold methods often estimate significance for variants individually.

As in the common theme of Bayesian shrinkage regression methods, our knowledge on variant effect appears only in its prior variance, since all effects are shrunk toward a single prior mean, which is zero. As such, the weight only indicates magnitude of variant effect but not direction. Indeed, for variants with high phastCons weights, it is unlikely for their minor alleles to have a protective role in disease liability. Thus, it is desirable to assign a positive prior mean for these variants in order to enhance the power of discovery. On the other hand, for variants with near zero weights, we still can use zero mean as prior. These considerations require a different prior specification than presented here as well as more complicated posterior computations [40], which will be explored in further extension of the present work.

An alternative Bayesian approach to identifying variants associated with disease is Bayesian model selection via Bayes factor. A representative example under this framework is the Bayesian risk index method [41], which allows for uncertainty of inclusion of variants and the direction of the effect. As opposed to fixing the number of variants, this feature offers the method some advantage when the proportion of causal variants is low in a region under study. Specifically, it provides a global evidence of a set of variants for their association with the disease and, if there is a global association, it furthers asks which variants are driving the association. Our method differs from the Bayesian risk index in the following essential ways. First, in our multi-variant regression model, a fully Bayesian treatment is used for the estimation of variant effects, whereas the computation of Bayes factor (as in Bayesian risk index) relies on approximation of marginal likelihood using maximum likelihood estimates of variant effects. That is, our method captures the uncertainty of variant effects through prior distributions. Second, variant-specific weights can be readily incorporated in our regression model, which has not been made possible in the Bayesian risk index. Furthermore, Bayes factor is a quantity that evaluates evidence in favor of a specific model; typically a value greater than 3.2 is interpreted as positive support. However, deciding upon a significant threshold for Bayes factor and thereby making decision is non-trivial and requires assumptions such as the relative cost of type II error to type I error [42]. Hence, our choice of using a test statistic followed by permutation test to control family wise type I error appears to be more convenient for declaring significance of a variant.

We tested our method on a relative small candidate region (1 Mb) around GWAS signals for convenient demonstration of the method, mainly for computational feasibility as many replicates needed to be run in the simulation study. The method can be extended to a larger candidate region to account for the situation that some causal variants could be far away from GWAS signals. Also, while we focused on low frequency candidate variants in our analyses, the method can readily encompass both common and rare variations. In both cases where the number of variants to be fitted in the model would be increased substantially, sample size needs to be increased accordingly to ensure accurate estimation of variant effects. On the other hand, as computing time grows when a large number of variants are analyzed, it may be useful to prescreen variants based on one or more criteria (e.g., exonic versus intergenic). In addition, another incentive for prescreening is that, with a limited sample size, adding more non-causal variants to the model would lead to less accurate effect size estimation for causal variants. In fact, the same argument holds for single variant test where prescreening can alleviate the burden of multiple testing.

A common problem with a multiple regression model is multicollinearity among variables. In our context, this is caused by high LD between variants. In particular, variances of effect estimates increase for variants that are in LD with at least one other variant in the same model. This often leads to false rejection of a true association. In our method, this problem is mitigated by differential shrinkage on variant effects through differential weighting. That is, despite being highly correlated in their genotypes, variants in high LD can still be distinguished by additional information such as the phastCons score. This may explain why the supplement of phastCons to ‘*r* weight’ improved power substantially. We also performed a small experiment (Text S3) where highly correlated variants were clustered and tagged. We tested the ability of the Bayesian model with ‘no weight’ to detect variant clusters that contained the causal variant as opposed to its ability to detect individual causal variants without clustering and tagging. Interestingly, clustering improved the power from 56% to 96% for causal MAF between 4–5%. However, the resolution to individual variants was lost by clustering and tagging. Nonetheless, when an informative weight is not available, this provides an efficient way to narrow down to variant clusters that contain causal variants.

Our method is implemented to be able to include covariates such as principal components of SNP genotypes. However, an open issue exists with the permutation procedure in its validity in the presence of confounding covariates. In this case, naive shuffling of disease statuses would break the confounding structure observed in the original data. There are existing methods that can effectively preserve relationships between confounding covariates and genotypes as well as between covariates and disease statuses [43].

## Supporting Information

Figure S1 Upper: pairwise LD (*r* ^2^) of the 100 candidate variants in *NOD2* region in the exome sequencing data. Lower: LD (*r*) between each candidate variant and the GWAS signal. Causal variants are marked in red color.
(PDF)

Figure S2 Same as Figure S1 but for the 338 candidate variants in *ITPA* region.
(PDF)

Figure S3 Distributions of the simulated liability values for exome sequencing samples. Dashed line marks median of the distribution of all samples, which was used to classify samples to cases and controls. (A): *NOD2* data, where 12% of samples were risk allele carriers. (B): *ITPA* data, where 39% of samples were risk allele carriers.
(PDF)

Figure S4 Heatmap plots showing the number of non-causal variants that were falsely identified as causal in each of the 200 replicates. Numbers on the right side of each plot are false positive rates averaged across replicates for different methods.
(PDF)

Figure S5 Distributions of standardized variant effect estimates from Bayesian liability model with phastCons weight and with *r*×phastCons weight. In each scenario (plot), effect estimates of Bayesian with phastCons weight (or *r*×phastCons) were collected from 200 replicates to form a distribution density.
(PDF)

Figure S6 Same as Figure 2 except that weight is shown on the log10 scale.
(PDF)

Text S1 Gibbs sampling in Bayesian liability regression model.
(PDF)

Text S2 Exome sequencing data processing and quality control.
(PDF)

Text S3 Using simulation to demonstrate effect of clustering variants on power.
(PDF)
