# Supplementary material for: Leveraging Prior Information to Detect Causal Variants via Multi-Variant Regression
Source: PLoS Comput Biol. 2013 Jun 6;9(6):e1003093. doi: 10.1371/journal.pcbi.1003093 (PMC3675126; doi:10.1371/journal.pcbi.1003093)
Supplement: Figure S1 — Upper: pairwise LD (r 2) of the 100 candidate variants in NOD2 region in the exome sequencing data. Lower: LD (r) between each candidate variant and the GWAS signal. Causal variants are marked in red color. (PDF) [file pcbi.1003093.s001.pdf]

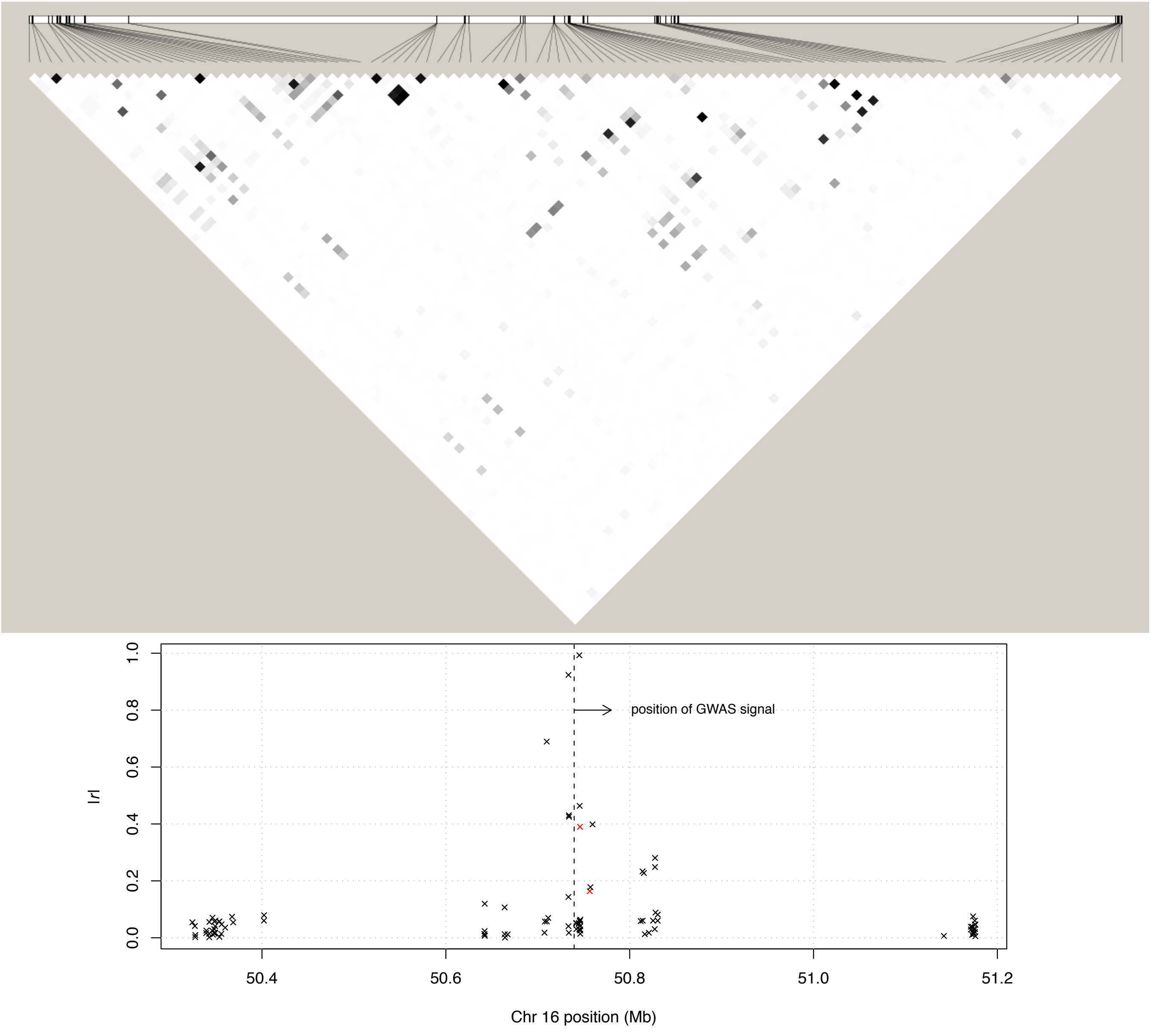

Figure S 1: Upper: pairwise LD (r^2) of the 100 candidate variants in *NOD2* region in the exome sequencing data. Lower: LD (r) between each candidate variant and the GWAS signal. Causal variants are marked in red color.
