# Supplementary figures and images for: Leveraging Prior Information to Detect Causal Variants via Multi-Variant Regression
Source: PLoS Comput Biol. 2013 Jun 6;9(6):e1003093. doi: 10.1371/journal.pcbi.1003093 (PMC3675126; doi:10.1371/journal.pcbi.1003093)

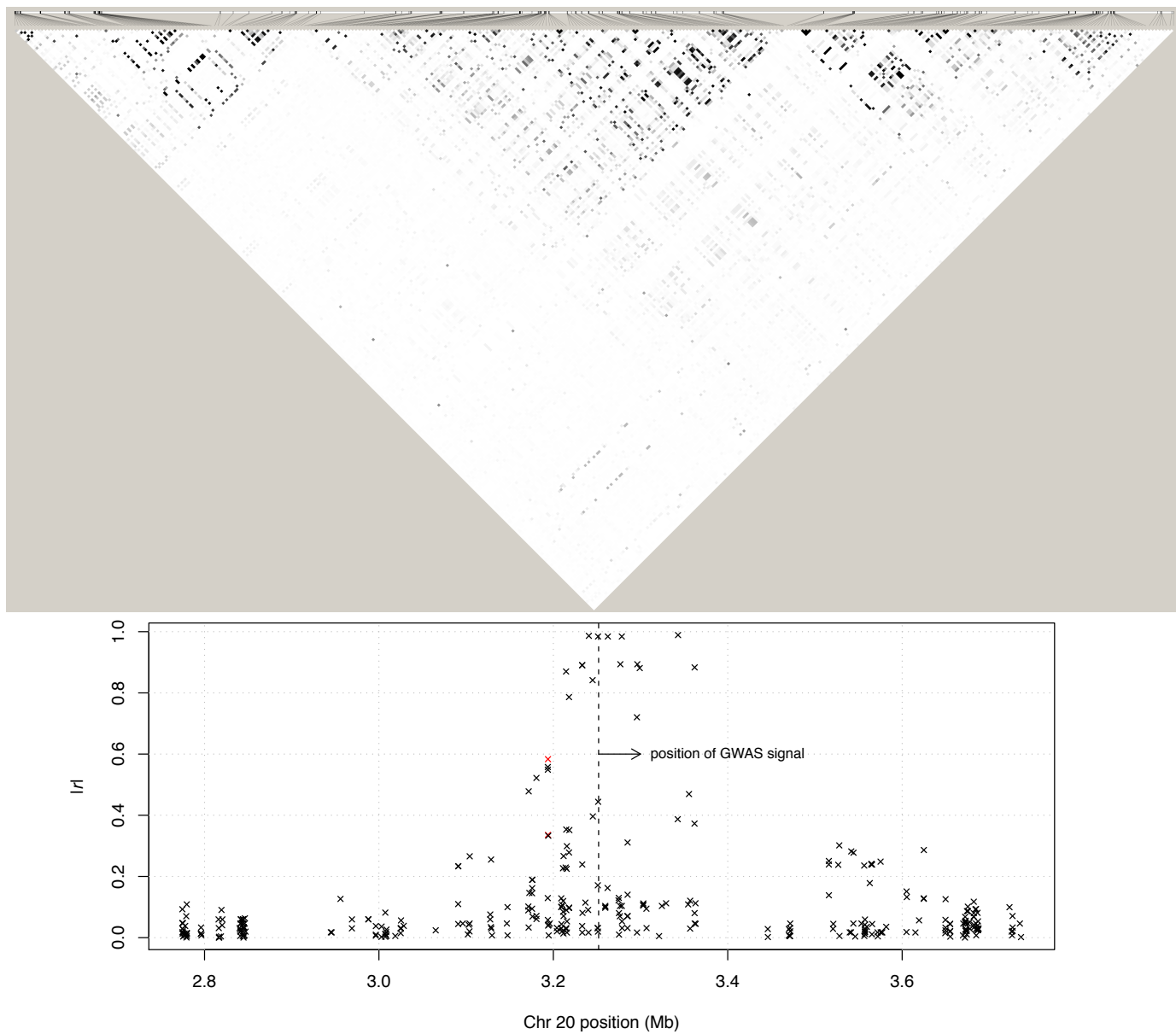

Figure S 2: Same as Figure S1 but for the 338 candidate variants in *ITPA* region.

Supplement: Figure S2 — Same as Figure S1 but for the 338 candidate variants in ITPA region. (PDF) [file pcbi.1003093.s002.pdf]

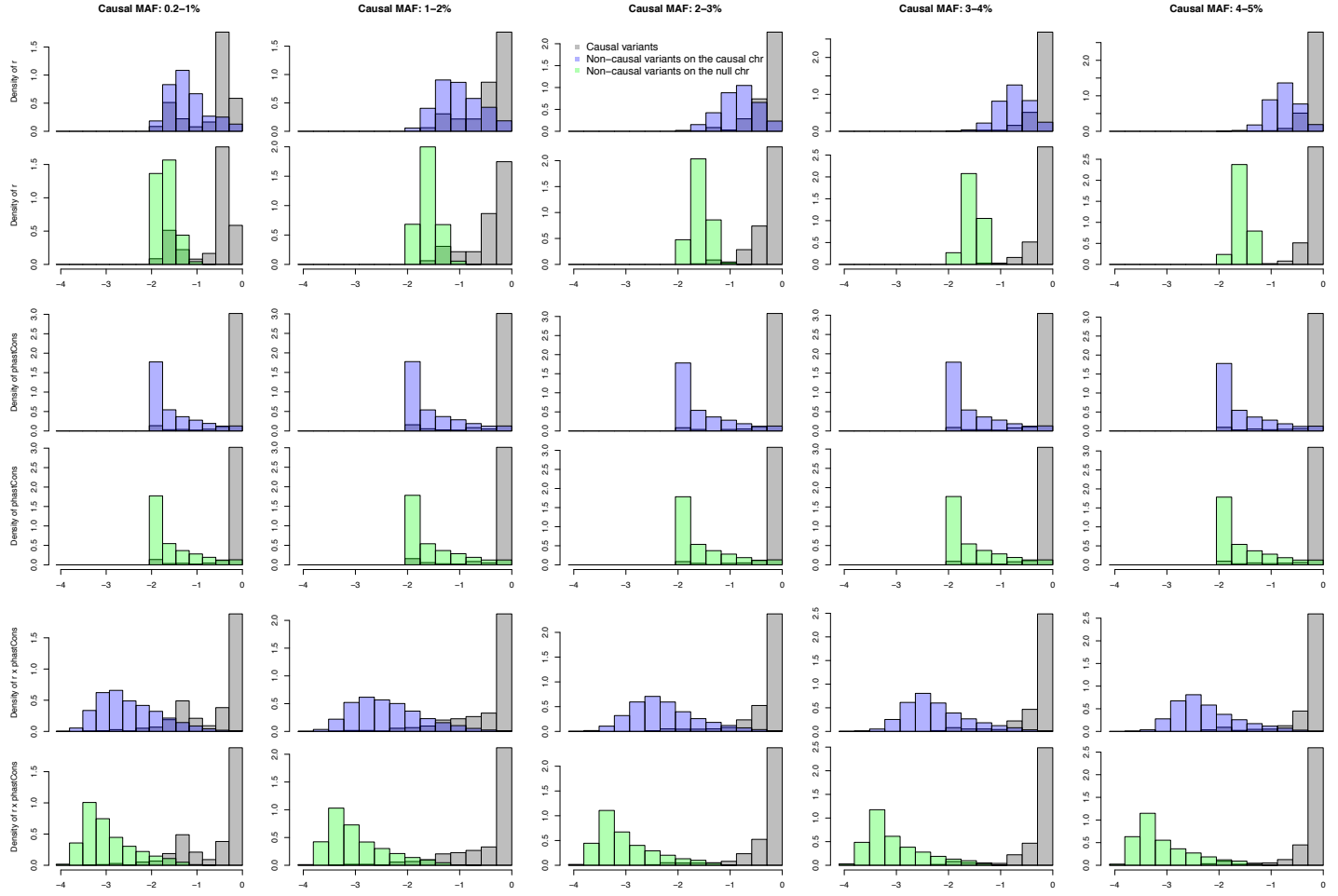

Figure S 6: Same as Figure 2 except that weight is shown on the  $\log_{10}$  scale.

Supplement: Figure S6 — Same as Figure 2 except that weight is shown on the log10 scale. (PDF) [file pcbi.1003093.s006.pdf]
