# Supplementary material for: Leveraging Prior Information to Detect Causal Variants via Multi-Variant Regression
Source: PLoS Comput Biol. 2013 Jun 6;9(6):e1003093. doi: 10.1371/journal.pcbi.1003093 (PMC3675126; doi:10.1371/journal.pcbi.1003093)
Supplement: Figure S3 — Distributions of the simulated liability values for exome sequencing samples. Dashed line marks median of the distribution of all samples, which was used to classify samples to cases and controls. (A): NOD2 data, where 12% of samples were risk allele carriers. (B): ITPA data, where 39% of samples were risk allele carriers. (PDF) [file pcbi.1003093.s003.pdf]

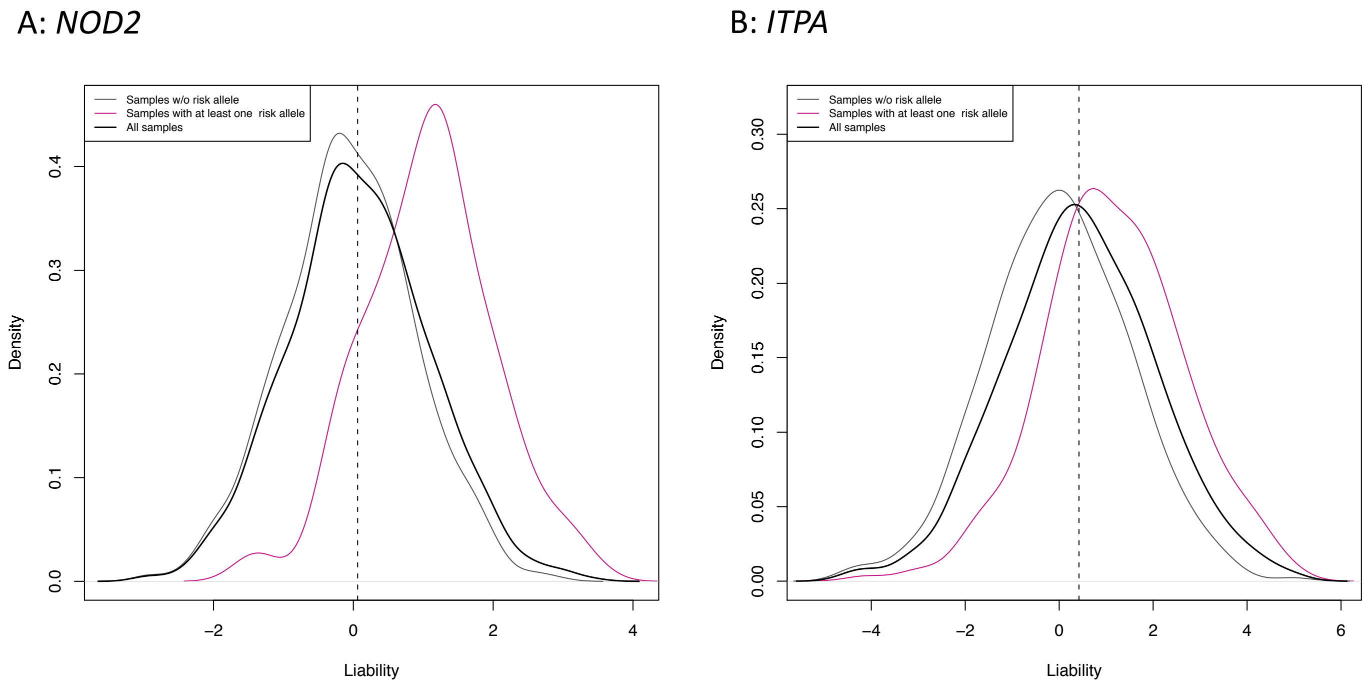

Figure S 3: Distributions of the simulated liability values for exome sequencing samples. Dashed line marks median of the distribution of all samples, which was used to classify samples to cases and controls. (A): *NOD2* data, where 12% of samples were risk allele carriers. (B): *ITPA* data, where 39% of samples were risk allele carriers.
