# Supplementary material for: Leveraging Prior Information to Detect Causal Variants via Multi-Variant Regression
Source: PLoS Comput Biol. 2013 Jun 6;9(6):e1003093. doi: 10.1371/journal.pcbi.1003093 (PMC3675126; doi:10.1371/journal.pcbi.1003093)
Supplement: Figure S4 — Heatmap plots showing the number of non-causal variants that were falsely identified as causal in each of the 200 replicates. Numbers on the right side of each plot are false positive rates averaged across replicates for different methods. (PDF) [file pcbi.1003093.s004.pdf]

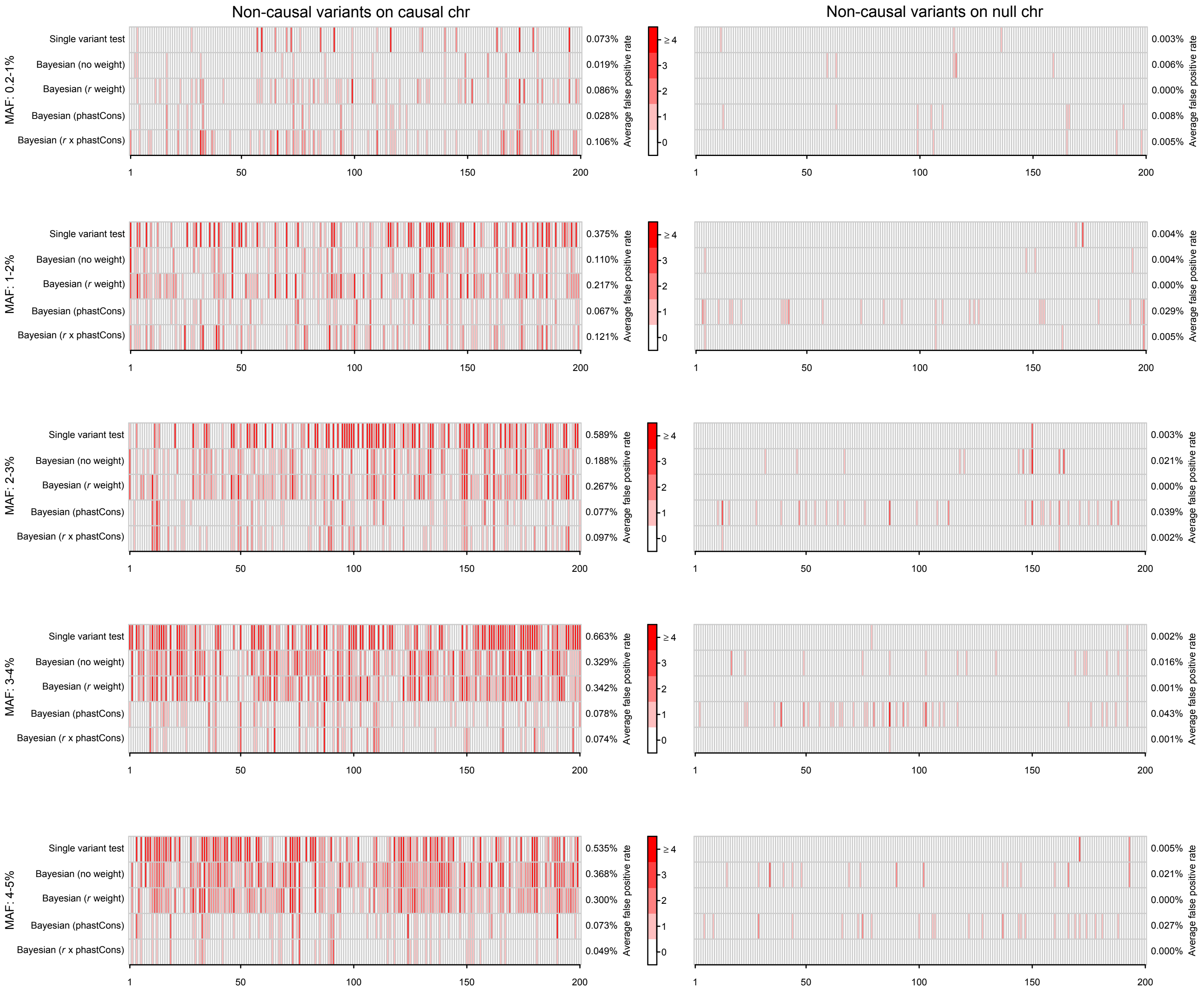

Figure S 4: Heatmap plots showing the number of non-causal variants that were falsely identified as causal in each of the 200 replicates. Numbers on the right side of each plot are false positive rates averaged across replicates for different methods.
