# Supplementary material for: Leveraging Prior Information to Detect Causal Variants via Multi-Variant Regression
Source: PLoS Comput Biol. 2013 Jun 6;9(6):e1003093. doi: 10.1371/journal.pcbi.1003093 (PMC3675126; doi:10.1371/journal.pcbi.1003093)
Supplement: Figure S5 — Distributions of standardized variant effect estimates from Bayesian liability model with phastCons weight and with r×phastCons weight. In each scenario (plot), effect estimates of Bayesian with phastCons weight (or r×phastCons) were collected from 200 replicates to form a distribution density. (PDF) [file pcbi.1003093.s005.pdf]

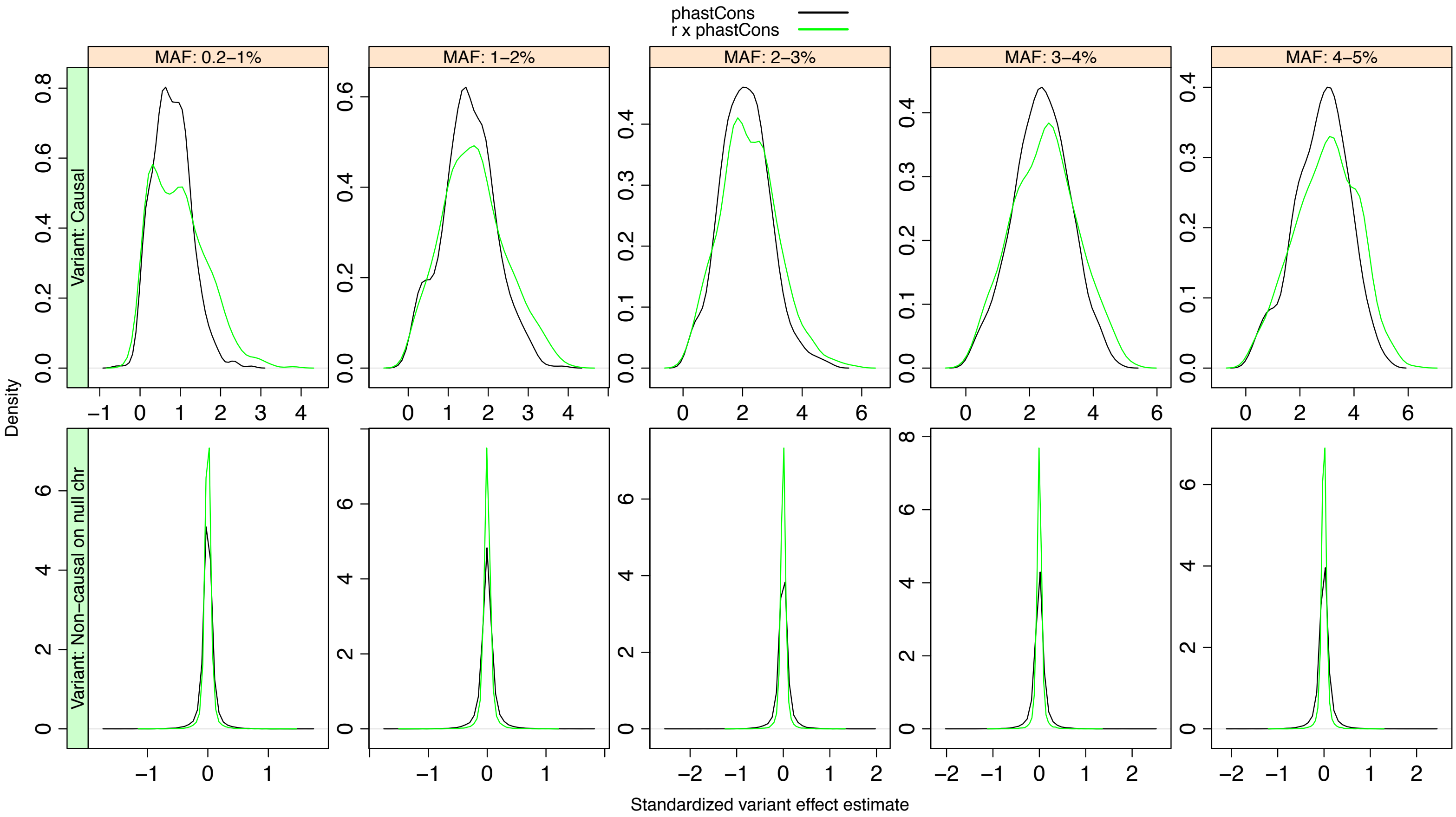

Figure S 5: Distributions of standardized variant effect estimates from Bayesian liability model with phastCons weight and with $r\times$phastCons weight. In each scenario (plot), effect estimates of Bayesian with phastCons weight (or $r\times$phastCons) were collected from 200 replicates to form a distribution density.
