# Supplementary material for: Leveraging Prior Information to Detect Causal Variants via Multi-Variant Regression
Source: PLoS Comput Biol. 2013 Jun 6;9(6):e1003093. doi: 10.1371/journal.pcbi.1003093 (PMC3675126; doi:10.1371/journal.pcbi.1003093)
Supplement: Text S1 — Gibbs sampling in Bayesian liability regression model. (PDF) [file pcbi.1003093.s007.pdf]

# Text S1

## Gibbs sampling in Bayesian liability regression model

Gibbs sampling is based on the fully conditional distribution of each unknown variable. This procedure iteratively goes through all variables, each time sampling from the distribution of one of them while fixing all others at their latest values. For liability modeling, the following steps are carried out (using notations in main text):

Start with arbitrary values for $\mu^{(t=0)}$, $\beta_j^{(t=0)}(j=1,\ldots,p)$, $\sigma^{2(t=0)}$

At iteration $t$+1:

1. Generate liability $y_i^{(t+1)}(i=1,\ldots,n)$ from a truncated Normal distribution, i.e., for $d_i$ = 1 sample $y_i$ from $N\left(\mu^{(t)}+\sum_{j=1}^{p}x_{ij}\beta_j^{(t)},1\right)$ until $y_i > 0$; similarly, for $d_i$ = 0 sample $y_i$ from $N\left(\mu^{(t)}+\sum_{j=1}^{p}x_{ij}\beta_j^{(t)},1\right)$ until $y_i < 0$.
2. Generate $\mu^{(t+1)}$ from $N\left(\frac{1}{n}\sum_{i=1}^{n}\left(y_i^{(t)}-\sum_{j=1}^{p}x_{ij}\beta_j^{(t)}\right),\ \frac{1}{n}\right)$.
3. Generate $\beta_j$'s. $\beta_j^{(t+1)}$ is drawn from

$$N\left(\left(\sum_{i=1}^{n}x_{ij}^2+\left(w_j\sigma^{2(t)}\right)^{-1}\right)^{-1}\sum_{i=1}^{n}x_{ij}\left(y_i^{(t+1)}-\mu^{(t+1)}-\sum_{k<j}x_{ik}\beta_k^{(t+1)}-\sum_{k>j}x_{ik}\beta_k^{(t)}\right),\ \left(\sum_{i=1}^{n}x_{ij}^2+\left(w_j\sigma^{2(t)}\right)^{-1}\right)^{-1}\right)$$

4. Generate $\sigma^{2(t+1)}$ from a scaled inverted Chi-square $\left(\sum_{j=1}^{p}\frac{\beta_j^{2(t+1)}}{w_j}+\nu S^2\right)\chi_{(\nu+p)}^{-2}$.
