# Supplementary material for: Leveraging Prior Information to Detect Causal Variants via Multi-Variant Regression
Source: PLoS Comput Biol. 2013 Jun 6;9(6):e1003093. doi: 10.1371/journal.pcbi.1003093 (PMC3675126; doi:10.1371/journal.pcbi.1003093)
Supplement: Text S2 — Exome sequencing data processing and quality control. (PDF) [file pcbi.1003093.s008.pdf]

Text S2

Exome sequencing data processing and quality control

Exome sequence reads of 753 individuals were aligned to NCBI Build 37 of human reference genome using BWA [1]. SAMtools [2] was used for variant calling. Only variants with Phred-scale quality score ≥ 25 and read depth ≥ 8 were kept. In the case of no variant called at a site for an individual, we set the genotype as homozygous reference allele if read depth at that site ≥ 8, and as missing otherwise.

For *NOD2* data, we focused on the +/- 500 Kb region around rs17221417 (the GWAS signal), which contained 608 SNPs. For this candidate region, we further applied the following quality control (QC) criteria: 1) missing rate per SNP ≤ 0.15; 2) missing rate per individual ≤ 0.1; 3) Hardy-Weinberg Equilibrium p value > 10^{-5}; and 4) MAF ≥ 0.2%. Finally, we used BEAGEL [3] (with default parameters) to impute the remaining sporadically missing genotypes and retained SNPs with allelic $R^2 \geq 0.9$. After QC, 728 samples and 100 SNPs genotyped on each were kept for statistical analysis.

For *ITPA* data, we focused on the +/- 500 Kb region around rs6051702 (the GWAS signal), which contained 1353 SNPs. Applying the same QC as above, we obtained 715 samples and 338 SNPs genotyped on each.

1. Li H, Durbin R (2009) Fast and accurate short read alignment with Burrows-Wheeler transform. Bioinformatics 25: 1754-1760.
2. Li H, Handsaker B, Wysoker A, Fennell T, Ruan J, et al. (2009) The Sequence Alignment/Map format and SAMtools. Bioinformatics 25: 2078-2079.
3. Browning BL, Browning SR (2007) Rapid and accurate haplotype phasing and missing data inference for whole genome association studies using localized haplotype clustering. Genetic Epidemiology 31: 606-606.
4. Ao SI, Yip K, Ng M, Cheung D, Fong PY, et al. (2005) CLUSTAG: hierarchical clustering and graph methods for selecting tag SNPs. Bioinformatics 21: 1735-1736.
