# Supplementary material for: Leveraging Prior Information to Detect Causal Variants via Multi-Variant Regression
Source: PLoS Comput Biol. 2013 Jun 6;9(6):e1003093. doi: 10.1371/journal.pcbi.1003093 (PMC3675126; doi:10.1371/journal.pcbi.1003093)
Supplement: Text S3 — Using simulation to demonstrate effect of clustering variants on power. (PDF) [file pcbi.1003093.s009.pdf]

Text S3

Using simulation to demonstrate effect of clustering variants on power

The simulation aimed to show that, in the absence of informative weight, we may improve power by clustering variants in high LD with each other. For convenience of investigation, we simulated one chromosome of 500 Kb length, which carried only one causal variant. The causal variant's MAF was between 4% to 5% and GRR was 3. The procedure was repeated 200 times and in each replication we required the causal variant be in high LD with at least one other variant; by high LD we meant $r^2 \geq 0.7$. We used a software called CLUSTAG [4] to perform clustering among variants, with r^2 cut off being 0.7. For each cluster, its tag SNP was chosen by the minimax algorithm implemented in CLUSTAG. Then we fit a Bayesian liability regression model with 'no weight' on all tag SNPs and followed the same significance test procedure as in the main analyses. Power in this case was assessed with respect to the 'causal cluster', the cluster of which the casual variant is a member.

1. Li H, Durbin R (2009) Fast and accurate short read alignment with Burrows-Wheeler transform. Bioinformatics 25: 1754-1760.
2. Li H, Handsaker B, Wysoker A, Fennell T, Ruan J, et al. (2009) The Sequence Alignment/Map format and SAMtools. Bioinformatics 25: 2078-2079.
3. Browning BL, Browning SR (2007) Rapid and accurate haplotype phasing and missing data inference for whole genome association studies using localized haplotype clustering. Genetic Epidemiology 31: 606-606.
4. Ao SI, Yip K, Ng M, Cheung D, Fong PY, et al. (2005) CLUSTAG: hierarchical clustering and graph methods for selecting tag SNPs. Bioinformatics 21: 1735-1736.
